# Crystal structure of chlorido­(η^2^-phenyl iso­thio­cyanate-κ^2^ *C*,*S*)-*mer*-tris­(tri­methyl­phosphane-κ*P*)iridium(I)

**DOI:** 10.1107/S160053681402162X

**Published:** 2014-10-18

**Authors:** Joseph S. Merola, Arthur W. Grieb

**Affiliations:** a Department of Chemistry 0212, Virginia Tech, Blacksburg, VA 24061, USA

**Keywords:** crystal structure, iridium complex, phenyl iso­thio­cyanate

## Abstract

The molecule of the title compound, [IrCl(C_7_H_5_NS)(C_3_H_9_P)_3_], is a distorted octa­hedral iridium complex with three PMe_3_ ligands arranged in a meridional geometry, a chloride ion *cis* to all three PMe_3_ groups and the phenyl iso­thio­cyanate ligand bonded in an η^2^-fashion through the C and S atoms. The C atom is *trans* to the chloride ion and the S atom is responsible for a significant deviation from an ideal octa­hedral geometry. The geometric parameters for the metal-complexing phenyl isothiocyanate group are compared with other metal-complexed phenyl iso­thio­cyanates, as well as with examples of uncomplexed aryl iso­thio­cyanates.

## Abstract 2

In this distorted octa­hedral iridium complex, the three PMe3 ligands are arranged in a meridional geometry, with the chloride ion *cis* to all three PMe3 groups and the phenyl iso­thio­cyanate ligand bonded in an η^2^-fashion through the C and S atoms. The geometric parameters for the metal-complexed PhNCS group are compared with other metal-complexed phenyl iso­thio­cyanates, as well as with examples of uncomplexed aryl iso­thio­cyanates.

## Chemical context   

Various phenyl iso­thio­cyanate complexes of metals have been characterized, all showing the effect of complexation of lengthening of N—C and C—S bonds and the bending of the N—C—S angle away from linearity. Complexation of an aryl iso­thio­cyanate to a metal has a similar effect across a wide range of metal systems with the N—C bond length averaging about 1.26 Å, the C—S distance averaging about 1.74 Å and the N—C—S bond angle ranging from 137 to 142°.
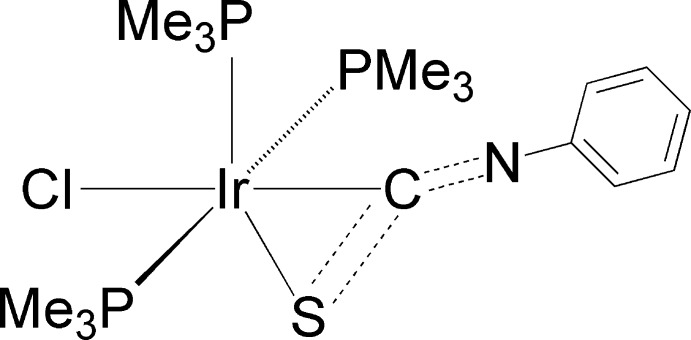


## Structural commentary   

**Figure 1:**
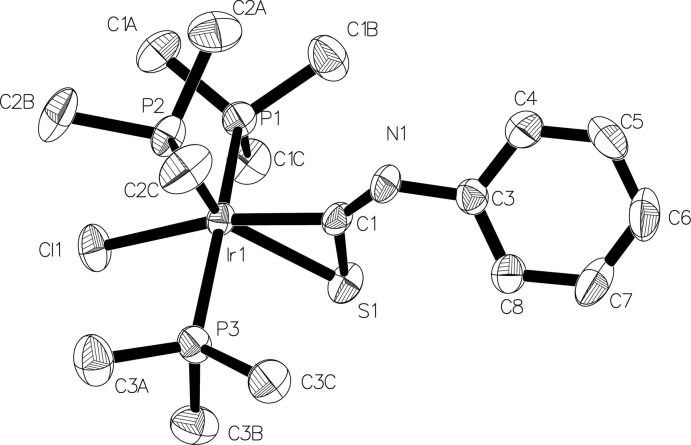
Displacement ellipsoid drawing of the title compound. Ellipsoids are drawn at the 50% probability level and hydrogen atoms are omitted for clarity.

The molecule of the title iridium compound has a distorted octa­hedral coordination sphere with three PMe_3_ ligands arranged in a meridional geometry, a chloride ion *cis* to all three PMe_3_ groups and the phenyl iso­thio­cyanate bonded in an η^2^ fashion to the C and S atoms (Fig. 1 ▶). The C atom is *trans* to the chloride ion and the S atom is significantly off from an ideal octa­hedral geometry [the P2—Ir1—S1 angle is 144.51 (5)° instead of the expected angle near 180°].

Upon complexation to the iridium cation in the title compound, the N—C bond in phenyl iso­thio­cyanate lengthens to 1.256 (7) Å, the C—S bond lengthens to 1.757 (6) Å and the N—C—S bond angle bends to 137.2 (4)°. These significant changes in geometry reflect the normal consequences of π-bonding of the C–S π-electrons to the metal and π-back-bonding from the metal to the π*-orbitals of the ligand.

## Database survey   

A search of the Cambridge Crystallographic Database (Groom & Allen, 2014 ▶) on 28 January 2014 found 16 aryl iso­thio­cyanates in which the SCN group is not disordered on coordinating to a metal. All of those structures display a nearly linear N—C—S geometry (ranging from 174–179° with an average of 176°). The multiply bonded nature of both the C—S and C—N bonds is seen in the bond lengths. For C—N, the distances range from 1.14 to 1.17 Å with an average of 1.16 Å and the C—S distances range from 1.54 to 1.59 Å with an average of 1.57 Å. Of those 16, four structures of good precision with no disorder, ionic inter­actions or other complex inter­actions that could affect the geometry of the N—C—S group were chosen for comparison to contrast ‘free’ *versus* ‘complexed’ iso­thio­cyanates. The first entry in Table 1 ▶ shows the average values for all 16 structures, the next four entries are the specific non-complexed aryl iso­thio­cyanates, the next six entries are other examples from the CCDC in which phenyl iso­thio­cyanate is complexed to a metal and the last entry is the data from the title compound. For the structures of several uncomplexed aryl iso­thio­cyanates, see: Majewska *et al.* (2007 ▶, 2008 ▶); Laliberté *et al.* (2004 ▶); Biswas *et al.* (2007 ▶). For the structures of a cobalt and a nickel complex of phenyl iso­thio­cyanate, see: Bianchini *et al.* (1984 ▶). For the structure of a vanadium complex of phenyl iso­thio­cyanate see: Gambarotta *et al.* (1984 ▶). For a phenyl iso­thio­cyanate complex of molybdenum, see: Ohnishi *et al.* (2005 ▶). For a phenyl iso­thio­cyanate complex of osmium, see: Flügel *et al.* (1996 ▶). For a tris-tri­methyl­phosphine nickel complex of phenyl iso­thio­cyanate, see: Huang *et al.* (2013 ▶).

## Synthesis and crystallization   

The crystal used in this experiment was obtained from a reaction between [Ir(COD)(PMe_3_)_3_]Cl (COD = 1,5-cyclo­octa­diene) and phenyl iso­thio­cyanate in toluene solution. Suitable single crystals were grown from di­chloro­methane by the layering of diethyl ether.

## Refinement   

Crystal data, data collection and structure refinement details are summarized in Table 2 ▶. H atoms were placed at calculated positions and refined using a model in which the hydrogen rides on the atom to which it is attached. For methyl hydrogen atoms *U*_iso_(H) = 1.5Ueq(C) and for the phenyl hydrogen atoms, *U*_iso_(H) = 1.2Ueq(C).

## Supplementary Material

Crystal structure: contains datablock(s) I. DOI: 10.1107/S160053681402162X/zl2576sup1.cif

Structure factors: contains datablock(s) I. DOI: 10.1107/S160053681402162X/zl2576Isup2.hkl

Supporting information file. DOI: 10.1107/S160053681402162X/zl2576Isup4.mol

CCDC reference: 1027097

Additional supporting information:  crystallographic information; 3D view; checkCIF report

## Figures and Tables

**Table 1:** Comparison of bond lengths and angles (, ) for the SCN moiety of isothiocyanate complexes

Compound	CCDC refcode	NC	CS	NCS	Reference
**Not complexing to a metal**					
Average of 16 compounds		1.16	1.57	176	Groom Allen (2014 ▶)
C_29_H_16_N_4_S_4_	221549	1.152(5)	1.566(4)	175.7(3)	Lalibert *et al.* (2004 ▶)
C_21_H_23_N_1_O_2_S_1_	673469	1.174(3)	!.584(3)	177.6(3)	Majewska *et al.* (2008 ▶)
C_24_H_37_N_1_S_1_	637960	1.134(7)	1.543(6)	176.1(5)	Biswas *et al.* (2007 ▶)
C_21_H_21_N_1_O_1_S_1_	646594	1.167(4)	1.587(4)	178.8(3)	Majewska *et al.* (2007 ▶)
					
**Complexing to a metal**					
C_48_H_44_N_1_Ni_1_P_3_S	555280	1.26(3)	1.68(3)	142(2)	Bianchini *et al.* (1984 ▶)
C_49_H_47_Co_1_N_2_P_3_S	555508	1.27(2)	1.72(1)	141(1)	Bianchini *et al.* (1984 ▶)
C_27_H_35_N_1_S_1_V_1_	557730	1.265(9)	1.745(7)	138.6(6)	Gambarotta *et al.* (1984 ▶)
C_70_H_63_Mo_1_N_3_P_4_S_2_	257394	1.256(7)	1.737(5)	134.9(4)	Ohnishi *et al.* (2005 ▶)
C_25_H_47_ClN_2_O_1_Os_1_P_2_S_1_	661980	1.253(7)	1.764(6)	141.2(4)	Flgel *et al.* (1996 ▶)
C_16_H_32_N_1_Ni_1_P_3_S	850129	1.253(3)	1.707(2)	142.2(2)	Huang *et al.* (2013 ▶)
C_16_H_32_Cl_1_Ir_1_N_1_P_3_S_1_	1027097	1.256(7)	1.757(6)	137.2(4)	This work

**Table 2:** Experimental details

Crystal data	
Chemical formula	[IrCl(C_7_H_5_NS)(C_3_H_9_P)_3_]
*M*_r_	591.05
Crystal system, space group	Monoclinic, *P*2_1_/*n*
Temperature (K)	293
*a*, *b*, *c* ()	8.964(2), 27.074(7), 9.721(2)
()	102.054(19)
*V* (^3^)	2307.3(10)
*Z*	4
Radiation type	Mo *K*
(mm^1^)	6.20
Crystal size (mm)	0.3 0.2 0.2

Data collection	
Diffractometer	Siemens P4
Absorption correction	scan (North *et al.*, 1968 ▶)
*T*_min_, *T*_max_	0.757, 0.891
No. of measured, independent and observed [*I* > 2(*I*)] reflections	5294, 5294, 4133
(sin /)_max_ (^1^)	0.650

Refinement	
*R*[*F* ^2^ > 2(*F* ^2^)], *wR*(*F* ^2^), *S*	0.031, 0.080, 0.93
No. of reflections	5294
No. of parameters	218
H-atom treatment	H-atom parameters constrained
_max_, _min_ (e ^3^)	1.00, 1.19

Computer programs: *XSCANS* (Siemens, 1994 ▶), *SHELXTL* and *SHELXS97* (Sheldrick, 2008 ▶), *SHELXL97* (Sheldrick, 2008 ▶), and *OLEX2* (Dolomanov *et al.*, 2009 ▶).

## supplementary crystallographic information

### Crystal data

**Table:**

[IrCl(C_7_H_5_NS)(C_3_H_9_P)_3_]	*F*(000) = 1160
*M**_r_* = 591.05	*D*_x_ = 1.701 Mg m^−^^3^
Monoclinic, *P*2_1_/*n*	Mo *K*α radiation, λ = 0.71073 Å
*a* = 8.964 (2) Å	Cell parameters from 45 reflections
*b* = 27.074 (7) Å	θ = 2–22°
*c* = 9.721 (2) Å	µ = 6.20 mm^−^^1^
β = 102.054 (19)°	*T* = 293 K
*V* = 2307.3 (10) Å^3^	Prism, yellow
*Z* = 4	0.3 × 0.2 × 0.2 mm

### Data collection

**Table:**

Siemens P4 diffractometer	4133 reflections with *I* > 2σ(*I*)
Radiation source: fine-focus sealed tube	*R*_int_ = 0.000
Graphite monochromator	θ_max_ = 27.5°, θ_min_ = 2.3°
ω scans	*h* = −11→11
Absorption correction: ψ scan (North *et al.*, 1968)	*k* = 0→35
*T*_min_ = 0.757, *T*_max_ = 0.891	*l* = 0→12
5294 measured reflections	2 standard reflections every 400 reflections
5294 independent reflections	intensity decay: 0.0(1)

### Refinement

**Table:**

Refinement on *F*^2^	Secondary atom site location: difference Fourier map
Least-squares matrix: full	Hydrogen site location: inferred from neighbouring sites
*R*[*F*^2^ > 2σ(*F*^2^)] = 0.031	H-atom parameters constrained
*wR*(*F*^2^) = 0.080	*w* = 1/[σ^2^(*F*_o_^2^) + (0.0433*P*)^2^] where *P* = (*F*_o_^2^ + 2*F*_c_^2^)/3
*S* = 0.93	(Δ/σ)_max_ = 0.002
5294 reflections	Δρ_max_ = 1.00 e Å^−^^3^
218 parameters	Δρ_min_ = −1.19 e Å^−^^3^
0 restraints	Extinction correction: *SHELXL97* (Sheldrick, 2008), Fc^*^=kFc[1+0.001xFc^2^λ^3^/sin(2θ)]^-1/4^
Primary atom site location: heavy-atom method	Extinction coefficient: 0.00040 (9)

### Special details

**Table:**

Geometry. All e.s.d.'s (except the e.s.d. in the dihedral angle between two l.s. planes) are estimated using the full covariance matrix. The cell e.s.d.'s are taken into account individually in the estimation of e.s.d.'s in distances, angles and torsion angles; correlations between e.s.d.'s in cell parameters are only used when they are defined by crystal symmetry. An approximate (isotropic) treatment of cell e.s.d.'s is used for estimating e.s.d.'s involving l.s. planes.
Refinement. Refinement of *F*^2^ against ALL reflections. The weighted *R*-factor *wR* and goodness of fit *S* are based on *F*^2^, conventional *R*-factors *R* are based on *F*, with *F* set to zero for negative *F*^2^. The threshold expression of *F*^2^ > σ(*F*^2^) is used only for calculating *R*-factors(gt) *etc*. and is not relevant to the choice of reflections for refinement. *R*-factors based on *F*^2^ are statistically about twice as large as those based on *F*, and *R*- factors based on ALL data will be even larger.

### Fractional atomic coordinates and isotropic or equivalent isotropic displacement parameters (Å^2^)

**Table:**

	*x*	*y*	*z*	*U*_iso_*/*U*_eq_	
Ir1	0.43752 (2)	0.345208 (7)	0.04153 (2)	0.02506 (7)	
Cl1	0.46057 (19)	0.27098 (5)	−0.10524 (16)	0.0442 (4)	
P1	0.39424 (18)	0.28837 (5)	0.20883 (16)	0.0331 (3)	
P2	0.68593 (17)	0.35657 (5)	0.13576 (17)	0.0352 (3)	
P3	0.43328 (19)	0.38958 (5)	−0.16484 (16)	0.0355 (3)	
S1	0.17603 (16)	0.37728 (5)	0.03289 (17)	0.0377 (3)	
C1A	0.5316 (8)	0.2394 (2)	0.2543 (7)	0.0543 (17)	
H1AA	0.5381	0.2210	0.1713	0.082*	
H1AB	0.5002	0.2178	0.3213	0.082*	
H1AC	0.6296	0.2532	0.2946	0.082*	
C1B	0.3641 (9)	0.3133 (2)	0.3741 (7)	0.0578 (19)	
H1BA	0.4540	0.3305	0.4207	0.087*	
H1BB	0.3430	0.2868	0.4328	0.087*	
H1BC	0.2792	0.3357	0.3562	0.087*	
C1C	0.2226 (7)	0.2537 (2)	0.1451 (7)	0.0500 (16)	
H1CA	0.1409	0.2759	0.1075	0.075*	
H1CB	0.1968	0.2352	0.2211	0.075*	
H1CC	0.2385	0.2314	0.0727	0.075*	
C2A	0.7354 (8)	0.3558 (3)	0.3284 (7)	0.060 (2)	
H2AA	0.6775	0.3806	0.3646	0.091*	
H2AB	0.8423	0.3624	0.3596	0.091*	
H2AC	0.7122	0.3239	0.3618	0.091*	
C2B	0.8222 (8)	0.3143 (3)	0.0871 (9)	0.065 (2)	
H2BA	0.8001	0.2813	0.1129	0.097*	
H2BB	0.9233	0.3232	0.1352	0.097*	
H2BC	0.8156	0.3159	−0.0127	0.097*	
C2C	0.7610 (8)	0.4167 (2)	0.1038 (8)	0.0579 (19)	
H2CA	0.7478	0.4219	0.0043	0.087*	
H2CB	0.8676	0.4181	0.1466	0.087*	
H2CC	0.7074	0.4418	0.1435	0.087*	
C3A	0.5891 (9)	0.3812 (3)	−0.2562 (8)	0.0586 (19)	
H3AA	0.6814	0.3943	−0.1998	0.088*	
H3AB	0.5660	0.3983	−0.3447	0.088*	
H3AC	0.6022	0.3466	−0.2723	0.088*	
C3B	0.2699 (8)	0.3716 (2)	−0.2991 (7)	0.0528 (17)	
H3BA	0.2793	0.3375	−0.3228	0.079*	
H3BB	0.2656	0.3916	−0.3813	0.079*	
H3BC	0.1783	0.3761	−0.2642	0.079*	
C3C	0.4144 (9)	0.4560 (2)	−0.1565 (7)	0.0536 (18)	
H3CA	0.3149	0.4641	−0.1412	0.080*	
H3CB	0.4276	0.4704	−0.2435	0.080*	
H3CC	0.4907	0.4688	−0.0805	0.080*	
C1	0.3493 (6)	0.39985 (18)	0.1318 (6)	0.0295 (11)	
N1	0.3916 (5)	0.43280 (15)	0.2224 (5)	0.0330 (10)	
C3	0.2897 (6)	0.4657 (2)	0.2672 (6)	0.0336 (12)	
C4	0.3135 (9)	0.4781 (2)	0.4081 (7)	0.0576 (19)	
H4	0.3936	0.4634	0.4710	0.069*	
C5	0.2239 (12)	0.5111 (3)	0.4568 (7)	0.086 (3)	
H5	0.2426	0.5181	0.5525	0.103*	
C6	0.1056 (10)	0.5344 (3)	0.3676 (8)	0.068 (2)	
H6	0.0451	0.5573	0.4018	0.081*	
C7	0.0792 (8)	0.5232 (2)	0.2275 (8)	0.0567 (19)	
H7	−0.0013	0.5382	0.1658	0.068*	
C8	0.1712 (7)	0.4895 (2)	0.1760 (7)	0.0448 (15)	
H8	0.1535	0.4829	0.0801	0.054*	

### Atomic displacement parameters (Å^2^)

**Table:**

	*U*^11^	*U*^22^	*U*^33^	*U*^12^	*U*^13^	*U*^23^
Ir1	0.02846 (11)	0.01590 (10)	0.03174 (11)	−0.00011 (9)	0.00841 (7)	−0.00018 (8)
Cl1	0.0563 (9)	0.0274 (7)	0.0530 (9)	−0.0037 (6)	0.0206 (7)	−0.0132 (6)
P1	0.0430 (8)	0.0220 (7)	0.0358 (8)	0.0002 (6)	0.0115 (6)	0.0035 (6)
P2	0.0300 (7)	0.0232 (7)	0.0522 (9)	−0.0020 (5)	0.0081 (7)	−0.0023 (6)
P3	0.0502 (9)	0.0229 (7)	0.0355 (8)	−0.0011 (6)	0.0140 (7)	0.0003 (6)
S1	0.0295 (7)	0.0297 (7)	0.0525 (9)	0.0006 (6)	0.0055 (6)	−0.0032 (6)
C1A	0.062 (4)	0.033 (3)	0.066 (4)	0.012 (3)	0.009 (4)	0.014 (3)
C1B	0.093 (6)	0.040 (4)	0.048 (4)	0.004 (4)	0.032 (4)	0.002 (3)
C1C	0.045 (4)	0.043 (4)	0.063 (4)	−0.008 (3)	0.015 (3)	0.006 (3)
C2A	0.053 (4)	0.064 (5)	0.055 (4)	−0.011 (4)	−0.011 (3)	−0.002 (4)
C2B	0.042 (4)	0.058 (5)	0.099 (6)	0.022 (3)	0.025 (4)	0.010 (4)
C2C	0.048 (4)	0.028 (3)	0.090 (5)	−0.012 (3)	−0.003 (4)	0.006 (3)
C3A	0.073 (5)	0.046 (4)	0.067 (5)	−0.001 (4)	0.039 (4)	0.002 (3)
C3B	0.068 (5)	0.043 (4)	0.044 (4)	−0.005 (3)	0.003 (3)	0.003 (3)
C3C	0.083 (5)	0.026 (3)	0.055 (4)	0.004 (3)	0.021 (4)	0.008 (3)
C1	0.033 (3)	0.021 (2)	0.035 (3)	0.006 (2)	0.007 (2)	0.003 (2)
N1	0.033 (2)	0.021 (2)	0.044 (3)	0.0041 (19)	0.007 (2)	−0.0039 (19)
C3	0.040 (3)	0.028 (3)	0.034 (3)	0.002 (2)	0.010 (2)	−0.001 (2)
C4	0.079 (5)	0.049 (4)	0.043 (4)	0.027 (4)	0.008 (4)	0.003 (3)
C5	0.138 (9)	0.090 (6)	0.034 (4)	0.049 (6)	0.029 (5)	−0.001 (4)
C6	0.084 (6)	0.055 (5)	0.073 (5)	0.028 (4)	0.036 (5)	−0.008 (4)
C7	0.043 (4)	0.042 (4)	0.080 (5)	0.016 (3)	0.002 (4)	−0.007 (4)
C8	0.057 (4)	0.033 (3)	0.042 (3)	0.010 (3)	0.006 (3)	−0.007 (3)

### Geometric parameters (Å, º)

**Table:**

Ir1—Cl1	2.4982 (14)	C2B—H2BA	0.9600
Ir1—P1	2.3297 (15)	C2B—H2BB	0.9600
Ir1—P2	2.2450 (16)	C2B—H2BC	0.9600
Ir1—P3	2.3319 (15)	C2C—H2CA	0.9600
Ir1—S1	2.4846 (15)	C2C—H2CB	0.9600
Ir1—C1	1.968 (5)	C2C—H2CC	0.9600
P1—C1A	1.801 (6)	C3A—H3AA	0.9600
P1—C1B	1.814 (6)	C3A—H3AB	0.9600
P1—C1C	1.799 (6)	C3A—H3AC	0.9600
P2—C2A	1.832 (7)	C3B—H3BA	0.9600
P2—C2B	1.808 (6)	C3B—H3BB	0.9600
P2—C2C	1.813 (6)	C3B—H3BC	0.9600
P3—C3A	1.819 (6)	C3C—H3CA	0.9600
P3—C3B	1.813 (7)	C3C—H3CB	0.9600
P3—C3C	1.810 (6)	C3C—H3CC	0.9600
S1—C1	1.757 (6)	C1—N1	1.256 (7)
C1A—H1AA	0.9600	N1—C3	1.408 (6)
C1A—H1AB	0.9600	C3—C4	1.383 (8)
C1A—H1AC	0.9600	C3—C8	1.391 (8)
C1B—H1BA	0.9600	C4—H4	0.9300
C1B—H1BB	0.9600	C4—C5	1.351 (9)
C1B—H1BC	0.9600	C5—H5	0.9300
C1C—H1CA	0.9600	C5—C6	1.375 (10)
C1C—H1CB	0.9600	C6—H6	0.9300
C1C—H1CC	0.9600	C6—C7	1.368 (10)
C2A—H2AA	0.9600	C7—H7	0.9300
C2A—H2AB	0.9600	C7—C8	1.391 (8)
C2A—H2AC	0.9600	C8—H8	0.9300
			
P1—Ir1—Cl1	85.01 (6)	H2AA—C2A—H2AB	109.5
P1—Ir1—P3	164.96 (6)	H2AA—C2A—H2AC	109.5
P1—Ir1—S1	87.77 (5)	H2AB—C2A—H2AC	109.5
P2—Ir1—Cl1	98.62 (5)	P2—C2B—H2BA	109.5
P2—Ir1—P1	95.81 (6)	P2—C2B—H2BB	109.5
P2—Ir1—P3	96.72 (6)	P2—C2B—H2BC	109.5
P2—Ir1—S1	144.51 (5)	H2BA—C2B—H2BB	109.5
P3—Ir1—Cl1	84.92 (5)	H2BA—C2B—H2BC	109.5
P3—Ir1—S1	86.85 (6)	H2BB—C2B—H2BC	109.5
S1—Ir1—Cl1	116.86 (5)	P2—C2C—H2CA	109.5
C1—Ir1—Cl1	161.48 (16)	P2—C2C—H2CB	109.5
C1—Ir1—P1	92.54 (16)	P2—C2C—H2CC	109.5
C1—Ir1—P2	99.88 (16)	H2CA—C2C—H2CB	109.5
C1—Ir1—P3	93.46 (15)	H2CA—C2C—H2CC	109.5
C1—Ir1—S1	44.63 (16)	H2CB—C2C—H2CC	109.5
C1A—P1—Ir1	116.9 (2)	P3—C3A—H3AA	109.5
C1A—P1—C1B	106.1 (3)	P3—C3A—H3AB	109.5
C1B—P1—Ir1	116.8 (2)	P3—C3A—H3AC	109.5
C1C—P1—Ir1	111.1 (2)	H3AA—C3A—H3AB	109.5
C1C—P1—C1A	101.0 (3)	H3AA—C3A—H3AC	109.5
C1C—P1—C1B	103.0 (3)	H3AB—C3A—H3AC	109.5
C2A—P2—Ir1	115.0 (3)	P3—C3B—H3BA	109.5
C2B—P2—Ir1	118.2 (3)	P3—C3B—H3BB	109.5
C2B—P2—C2A	103.2 (4)	P3—C3B—H3BC	109.5
C2B—P2—C2C	103.2 (3)	H3BA—C3B—H3BB	109.5
C2C—P2—Ir1	115.2 (2)	H3BA—C3B—H3BC	109.5
C2C—P2—C2A	99.6 (3)	H3BB—C3B—H3BC	109.5
C3A—P3—Ir1	118.7 (2)	P3—C3C—H3CA	109.5
C3B—P3—Ir1	110.3 (2)	P3—C3C—H3CB	109.5
C3B—P3—C3A	101.7 (4)	P3—C3C—H3CC	109.5
C3C—P3—Ir1	117.3 (2)	H3CA—C3C—H3CB	109.5
C3C—P3—C3A	103.5 (3)	H3CA—C3C—H3CC	109.5
C3C—P3—C3B	103.3 (3)	H3CB—C3C—H3CC	109.5
C1—S1—Ir1	51.90 (17)	S1—C1—Ir1	83.5 (2)
P1—C1A—H1AA	109.5	N1—C1—Ir1	139.2 (4)
P1—C1A—H1AB	109.5	N1—C1—S1	137.2 (4)
P1—C1A—H1AC	109.5	C1—N1—C3	123.1 (5)
H1AA—C1A—H1AB	109.5	C4—C3—N1	119.0 (5)
H1AA—C1A—H1AC	109.5	C4—C3—C8	117.2 (5)
H1AB—C1A—H1AC	109.5	C8—C3—N1	123.7 (5)
P1—C1B—H1BA	109.5	C3—C4—H4	119.1
P1—C1B—H1BB	109.5	C5—C4—C3	121.9 (6)
P1—C1B—H1BC	109.5	C5—C4—H4	119.1
H1BA—C1B—H1BB	109.5	C4—C5—H5	119.4
H1BA—C1B—H1BC	109.5	C4—C5—C6	121.3 (7)
H1BB—C1B—H1BC	109.5	C6—C5—H5	119.4
P1—C1C—H1CA	109.5	C5—C6—H6	120.8
P1—C1C—H1CB	109.5	C7—C6—C5	118.4 (6)
P1—C1C—H1CC	109.5	C7—C6—H6	120.8
H1CA—C1C—H1CB	109.5	C6—C7—H7	119.6
H1CA—C1C—H1CC	109.5	C6—C7—C8	120.8 (6)
H1CB—C1C—H1CC	109.5	C8—C7—H7	119.6
P2—C2A—H2AA	109.5	C3—C8—H8	119.8
P2—C2A—H2AB	109.5	C7—C8—C3	120.4 (6)
P2—C2A—H2AC	109.5	C7—C8—H8	119.8
			
Ir1—S1—C1—N1	−175.8 (7)	P3—Ir1—P2—C2B	−85.8 (3)
Ir1—C1—N1—C3	−176.7 (4)	P3—Ir1—P2—C2C	36.8 (3)
Cl1—Ir1—P1—C1A	52.0 (3)	P3—Ir1—S1—C1	−98.1 (2)
Cl1—Ir1—P1—C1B	179.2 (3)	P3—Ir1—C1—S1	81.98 (17)
Cl1—Ir1—P1—C1C	−63.2 (2)	P3—Ir1—C1—N1	−102.4 (6)
Cl1—Ir1—P2—C2A	−122.4 (3)	S1—Ir1—P1—C1A	169.2 (3)
Cl1—Ir1—P2—C2B	0.0 (3)	S1—Ir1—P1—C1B	−63.6 (3)
Cl1—Ir1—P2—C2C	122.6 (3)	S1—Ir1—P1—C1C	54.0 (2)
Cl1—Ir1—P3—C3A	−55.1 (3)	S1—Ir1—P2—C2A	57.8 (3)
Cl1—Ir1—P3—C3B	61.5 (3)	S1—Ir1—P2—C2B	−179.8 (3)
Cl1—Ir1—P3—C3C	179.3 (3)	S1—Ir1—P2—C2C	−57.2 (3)
Cl1—Ir1—S1—C1	179.2 (2)	S1—Ir1—P3—C3A	−172.4 (3)
Cl1—Ir1—C1—S1	−2.4 (6)	S1—Ir1—P3—C3B	−55.8 (3)
Cl1—Ir1—C1—N1	173.2 (4)	S1—Ir1—P3—C3C	62.0 (3)
P1—Ir1—P2—C2A	−36.6 (3)	S1—Ir1—C1—N1	175.6 (8)
P1—Ir1—P2—C2B	85.8 (3)	S1—C1—N1—C3	−3.2 (9)
P1—Ir1—P2—C2C	−151.5 (3)	C1—Ir1—P1—C1A	−146.4 (3)
P1—Ir1—P3—C3A	−103.2 (3)	C1—Ir1—P1—C1B	−19.2 (3)
P1—Ir1—P3—C3B	13.3 (3)	C1—Ir1—P1—C1C	98.4 (3)
P1—Ir1—P3—C3C	131.2 (3)	C1—Ir1—P2—C2A	57.1 (3)
P1—Ir1—S1—C1	95.9 (2)	C1—Ir1—P2—C2B	179.5 (3)
P1—Ir1—C1—S1	−84.23 (17)	C1—Ir1—P2—C2C	−57.9 (3)
P1—Ir1—C1—N1	91.4 (6)	C1—Ir1—P3—C3A	143.4 (3)
P2—Ir1—P1—C1A	−46.2 (3)	C1—Ir1—P3—C3B	−100.0 (3)
P2—Ir1—P1—C1B	81.0 (3)	C1—Ir1—P3—C3C	17.8 (3)
P2—Ir1—P1—C1C	−161.4 (2)	C1—N1—C3—C4	140.5 (6)
P2—Ir1—P3—C3A	43.0 (3)	C1—N1—C3—C8	−44.4 (8)
P2—Ir1—P3—C3B	159.6 (3)	N1—C3—C4—C5	177.1 (7)
P2—Ir1—P3—C3C	−82.6 (3)	N1—C3—C8—C7	−177.1 (6)
P2—Ir1—S1—C1	−1.0 (2)	C3—C4—C5—C6	−1.0 (14)
P2—Ir1—C1—S1	179.42 (14)	C4—C3—C8—C7	−1.9 (9)
P2—Ir1—C1—N1	−5.0 (6)	C4—C5—C6—C7	0.7 (14)
P3—Ir1—P1—C1A	100.1 (3)	C5—C6—C7—C8	−1.0 (12)
P3—Ir1—P1—C1B	−132.7 (3)	C6—C7—C8—C3	1.7 (11)
P3—Ir1—P1—C1C	−15.1 (3)	C8—C3—C4—C5	1.6 (11)
P3—Ir1—P2—C2A	151.8 (3)		
